# Unfolding and Folding of the Three-Helix Bundle Protein KIX in the Absence of Solvent

**DOI:** 10.1007/s13361-016-1363-7

**Published:** 2016-03-02

**Authors:** Moritz Schennach, Eva-Maria Schneeberger, Kathrin Breuker

**Affiliations:** Institute of Organic Chemistry and Center for Molecular Biosciences Innsbruck (CMBI), University of Innsbruck, Innrain 80/82, 6020 Innsbruck, Austria

**Keywords:** Electron capture dissociation, Gas phase, Native mass spectrometry, Protein, Protein folding

## Abstract

Electron capture dissociation was used to probe the structure, unfolding, and folding of KIX ions in the gas phase. At energies for vibrational activation that were sufficiently high to cause loss of small molecules such as NH_3_ and H_2_O by breaking of covalent bonds in about 5% of the KIX (M + nH)^n+^ ions with n = 7–9, only partial unfolding was observed, consistent with our previous hypothesis that salt bridges play an important role in stabilizing the native solution fold after transfer into the gas phase. Folding of the partially unfolded ions on a timescale of up to 10 s was observed only for (M + nH)^n+^ ions with n = 9, but not n = 7 and n = 8, which we attribute to differences in the distribution of charges within the (M + nH)^n+^ ions.

**Graphical Abstract:**
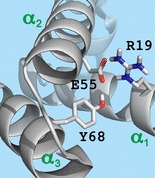
ᅟ

ᅟ

## Introduction

Native mass spectrometry (MS) has, over the past 25 years, developed from interpreting mass spectra from electrospray ionization (ESI) of different solutions to approaches by which the dissociation of biomolecules such as proteins and nucleic acids and their noncovalent complexes is studied, or their rotationally averaged collision cross section is probed by ion mobility MS [1–29]. Obtaining information of relevance to biological problems by native ESI MS relies, as a matter of course, on the preservation of solution structure after transfer into the gas phase, where it can be probed by a number of techniques, including electron capture dissociation (ECD) [30, 31]. However, the use of native ESI “is not without complications” [9], not least because the stability of a solution structure in the gas phase can presently not be reliably predicted. Nevertheless, some progress has been made in understanding the determinants of peptide and protein structure and stability in a gaseous environment [32–47].

We have previously postulated that salt bridges, either formed in the gas phase or already present in solution [48], can contribute substantially to the stabilization of the solution structure of a protein after transfer into the gas phase [33]. In support of this hypothesis, calculations suggest that in the absence of solvent, the strength of an overall neutral salt bridge can be comparable to the strength of a covalent bond [49, 50]. Ionic hydrogen bonds [48] between protonated basic or deprotonated acidic sites and neutral molecules have strengths of typically 21–146 kJ/mol, which is up to a third of the strength of covalent bonds [51]. Neutral hydrogen bonds between backbone amides constitute the basis of protein secondary structure (i.e., α-helices and β-sheets). The N-H⋅⋅⋅O=C hydrogen bond stability in the gas phase can be estimated to be close to the dimerization energy of two *N*-methylacetamide molecules of ~28 kJ/mol [52]. Although in the gas phase neutral hydrogen bonds are substantially weaker than both ionic hydrogen bonds and salt bridges, they are typically more numerous in proteins with a high content of secondary structure. Finally, ion–dipole interactions, especially those that involve helix dipole moments, can stabilize protein ion gas phase structure [33]. In the absence of sufficient stabilization by electrostatic interactions, desolvation can cause spontaneous unfolding of protein ions [53–57], which can subsequently fold into more stable gaseous ion structures [32]. However, only a small number of experimental studies [34, 58–61] have so far focused on peptide or protein folding in the gas phase, even though all possible structural transitions can affect data from native mass spectrometry experiments. We have recently reported that gaseous cytochrome *c* ions from horse and tuna heart, the fold of which is virtually identical in solution, show vastly dissimilar folding behavior, and found evidence that the formation of salt bridges is a major driving force for protein folding in the gas phase [34].

Here we investigate the unfolding and folding of the three-helix bundle protein KIX, for which ECD data indicated substantial preservation of the native solution structure in the (M + 7H)^7+^ ions, on a timescale of at least 4 s after transfer into the gas phase, even after vibrational ion activation by 28 eV collisions with argon gas [33]. In this study, we subjected KIX (M + nH)^n+^ ions with n = 7–9 to higher energy collisions that were sufficiently high to break covalent bonds in ~5% of the ion populations, and discuss the observed partial unfolding in terms of electrostatic interactions. Folding data for the partially unfolded KIX ions is complemented with data for a KIX peptide, which are discussed in the context of Coulombic repulsion and possible interactions that drive the folding process.

## Experimental

**Figure 1:**
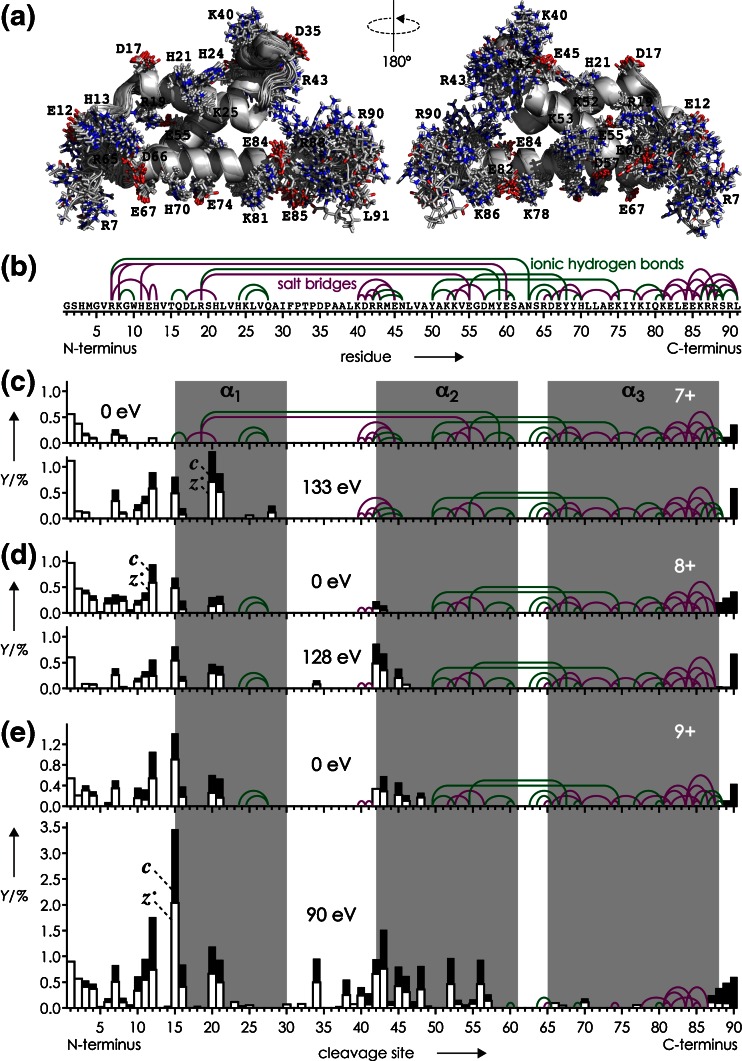
(**a**) Overlay of 20 KIX structures (pdb entry 2AGH) with basic (N-terminus, H, K, R) and acidic (C-terminus, D, E) residues shown as sticks (nitrogen: blue, oxygen: red); (**b**) possible salt bridges (SB, purple lines) and ionic hydrogen bonds (IHB, green lines); (**c**)–(**e**) yields of ***c*** (black bars) and ***z*** ^•^ (open bars) fragments from ECD of (M + nH)^n+^ ions of KIX for n = 7–9 as indicated, versus backbone cleavage site; data without collisional activation (upper traces, 0 eV) are from reference [33] and helix regions are shaded in gray; SB and IHB that were potentially preserved in each experiment are indicated

KIX protein (91 residues, GSHMGVRKGW HEHVTQDLRS HLVHKLVQAI FPTPDPAALK DRRMENLVAY AKKVEGDMYE SANSRDEYYH LLAEKIYKIQ KELEEKRRSR L) was expressed with an N-terminal hexahistidine (His_6_) tag in *Escherichia coli* cells and purified by Ni-affinity and size-exclusion chromatography (SEC); after removal of the His_6_ tag by incubation with thrombin and another purification step by SEC, KIX was desalted using centrifugal concentrators as described previously [33, 62, 63]. H_2_O was purified to 18 MΩ⋅cm at room temperature using a Milli-Q system (Millipore, Austria), CH_3_OH (Acros, Vienna, Austria) was HPLC-grade, and CH_3_COOH, CH_3_COONH_4_ (>99.0%, Na ≤5 mg/kg, K ≤5 mg/kg), and ethylenediamine diacetate (EDDA) were purchased from Sigma-Aldrich (Vienna, Austria). The KIX peptide comprising residues 36–91, KIX(36–91), was produced by acid hydrolysis of the desalted protein (9 μM in 97:3 H_2_O/CH_3_COOH, pH 2) at 99 °C for 2 h. The reaction was quenched by dilution to 2 mL in 80:20 H_2_O:CH_3_OH with 1 mM EDDA at room temperature. Under these conditions, >95% of the products were peptide KIX(36–91) and its complement comprising residues 1–35 from hydrolysis of the backbone amide bond between D35 and P36. Repetitive cycles of concentration using Vivaspin centrifugal concentrators (2 mL, molecular weight cut-off 2000; Sartorius, Austria) to ~200 μL and dilution in 80:20 H_2_O:CH_3_OH were performed until pH 4 was reached. As noted in our previous study [33], KIX tends to aggregate in unbuffered, aqueous solutions at pH 4.5–5.5, for which reason protein and peptide (M + nH)^n+^ ions were electrosprayed from 1–5 μM solutions in 80:20 H_2_O/CH_3_OH at pH 4 (adjusted by addition of acetic acid) with 1 mM EDDA [64], except for the folding experiments of KIX (M + 9H)^9+^ ions for which instead of EDDA, 500 μM ammonium acetate was used. The latter are chemically similar, organic salts comprising the same anion, acetate, which should not have significantly different effects on protein structure at the concentrations used [65, 66]. However, the use of EDDA instead of ammonium acetate produced higher yields of (M + 7H)^7+^ and (M + 8H)^8+^ ions [64]. Experiments were performed on a 7 T Fourier transform ion cyclotron resonance (FT-ICR) mass spectrometer (Bruker, Austria) equipped with an ESI source and a hollow dispenser cathode for ECD. For unfolding, protein (M + nH)^n+^ ions were vibrationally activated by energetic collisions in the ESI source region by application of a skimmer potential of 120 V for n = 7 and 80 V for n = 8 (5 V was used for n = 9), and by collisions with Ar gas (~10^−3^ mbar) at the head of the second hexapole at 19, 16, and 10 V potential for n = 7, 8, and 9, corresponding to 133, 128, and 90 eV laboratory frame energy, respectively. Unfolding of the (M + 6H)^6+^ ions of KIX(36–91) utilized 100 V skimmer and 15 V hexapole potentials. All potentials were optimized for maximum vibrational activation and structural annihilation without significant depletion of the ion population to subsequently undergo folding, which was realized by limiting the loss of small molecules (NH_3_, H_2_O etc.) from (M + nH)^n+^ ions to ~5%. Ions were accumulated in the first hexapole for <1 s, isolated by *m/z* in the quadrupole, accumulated in the second hexapole for <1 s, and transferred into the trapped ICR cell for ECD and ion detection; for a schematic of the experimental setup, see reference [67]. Folding was monitored by varying the delay between ion accumulation in the second hexapole and transfer (~2 ms) into the ICR cell from 0–10 s in 1 s intervals for KIX and 0–4 s in 0.5 s intervals for KIX(36–91). Five hundred scans were added for each spectrum. Site-specific fragment yields were calculated as %-values relative to all ECD products, considering that backbone cleavage gives a pair of complementary ***c*** and ***z***^•^ ions (***a***^•^, ***y*** ions were not included in the analysis because of their marginal abundance totaling to <1%): 100% = 0.5·[***c***] + 0.5·[***z***^•^] + [reduced molecular ions and loss of small neutral species from the latter]. Possible salt bridges and ionic hydrogen bonds in Figure 1b were identified by inspection of the 20 KIX structures in pdb entry 2AGH using the PyMOL molecular viewer (Schrödinger, LLC, NY, USA). A salt bridge was assigned when non-backbone nitrogen atoms of a basic (N-terminus, H, K, R) oxygen atoms of an acidic (C-terminus, D, E) residue were within 4 Å from each other, or when sidechain bonds could be rotated such that this distance constraint was reached. Similarly, ionic hydrogen bonds between basic or acidic and polar sidechains were assigned for heteroatom (N, O) distances <3 Å.

## Results and Discussion

The 20 KIX structures in pdb entry 2AGH, calculated on the basis of distance restraints from nuclear magnetic resonance (NMR) experiments in the Wright group [68], show a highly uniform backbone fold especially in the α-helical regions (α_1_: residues 16-30, α_2_: 42-61, α_3_: 65–88) but substantial variations in sidechain orientation of basic (H, K, R, N-terminus) and acidic (D, E, C-terminus) residues (Figure 1a). Other KIX structures (1KDX, 2LXS, 2LXT, 2LQH, 2LQI, 2KWF) exhibit essentially the same backbone fold and similarly large variations in sidechain orientation, notwithstanding the fact that all corresponding NMR experiments were conducted in the presence of different peptide ligands and under different solution conditions, i.e., different ionic strength (0–50 mM NaCl), buffers (Tris(hydroxymethyl)aminomethane acetate, potassium phosphate, 2-(*N*-morpholino)ethanesulfonic acid) at different concentrations (20–50 mM), and different pH values (5.5–6.0). Apparently, the conformational flexibility of KIX's basic and acidic sidechains is generally high in solution, although some electrostatic interactions such as the salt bridge between R19 and E55 (Figure 1a) were found in most of the above NMR structures.

We have previously proposed that the transfer of proteins into the gas phase by electrospray ionization causes the formation of salt bridges and ionic hydrogen bonds on the protein surface, by which a native fold can be stabilized during and after the phase transition [32, 48]. The high stability of KIX (M + 7H)^7+^ ions in the complete absence of solvent, on a timescale of up to 4 s [33], suggests that a sufficiently large number of electrostatic interactions have formed during ESI that, together with those already present in solution, prevent the native fold from disintegration in the gas phase. All salt bridges (SB, purple lines) and ionic hydrogen bonds (IHB, green lines) that are present in solution or can potentially form during ESI while retaining the backbone fold of the KIX structure 2AGH are illustrated in Figure 1b.

Comparing the possible salt bridges and ionic hydrogen bonds (Figure 1b) to the site-specific yields of ***c*** (black bars) and ***z***^•^ (open bars) fragments from ECD of KIX (M + 7H)^7+^ ions (Figure 1c, 0 eV) reveals that the possible interactions of R7, K8, H11, and H13 (SB: R7/E12, R7/E60, K8/E60, H11/E60, H13/E12, IHB: R7/N63, K8/W10) were, at least in a significant fraction of the ions, not present as this would have prevented separation of fragments from cleavage at sites 7, 8, and 12 [30]. However, after vibrational activation for unfolding of the KIX (M + 7H)^7+^ ions (Figure 1c, 133 eV), the yield of separated ***c*** and ***z***^•^ fragments from cleavage at sites 7 and 12 increased substantially, and fragments from sites 10 and 11 appeared, consistent with an increase in the fraction of ions in which the interactions of R7, K8, H11, and H13 were broken. Moreover, separated fragments were observed from cleavage in the region of helix α_1_ (sites 15, 16, 20, 21, 25, 28), indicating loss of its secondary structure along with breaking of any interactions between helices α_1_ and α_2_ (SB: R19/E55, IHB: R19/Y59). The ECD patterns of the KIX (M + 8H)^8+^ ions with and without collisional activation are very similar (Figure 1d), although breaking of SB and IHB interactions between residues 42, 43, 45, and 46 is evident from the ~4-fold increase in yield of fragments from sites 42, 43, and 45. However, collisional activation had a far stronger effect on the structure of the KIX (M + 9H)^9+^ ions, the fragmentation pattern of which at 0 eV (Figure 1e) was very similar to that of the (M + 8H)^8+^ ions at 128 eV. Specifically, the data indicate nearly full unraveling of helix α_2_ along with breaking of the IHB between helices α_2_ and α_3_ (Y50/K75, E55/Y68), and significant loss of residual structure in the region comprising residues 6–57 (note that ECD does not produce ***c*** and ***z***^•^ ions from cleavage at the N-terminal side of proline residues, which applies to sites 31, 33, and 35).

In summary, the above data suggest that unfolding of the three-helix bundle structure of the KIX (M + 7H)^7+^ ions by collisional activation, at energies that are sufficiently high to cause loss of small molecules such as NH_3_ and H_2_O by breaking of covalent bonds in about 5% of the ions, is limited to the separation and disruption of helix α_1_ while retaining the higher order structure of helices α_2_ and α_3_. By contrast, helix α_1_ is already unraveled and separated from helices α_2_ and α_3_ in a significant fraction of the (M + 8H)^8+^ ions produced by ESI, along with uncoiling of the first turn of helix α_2_, and collisional activation merely increases this fraction without causing additional structural changes. The structure of the (M + 9H)^9+^ ions from ESI is very similar to that of the partially unfolded (M + 8H)^8+^ ions, but after collisional activation, substantial loss of tertiary and secondary structure is observed, even though the latter is largely retained in helix α_3_.

**Figure 2:**
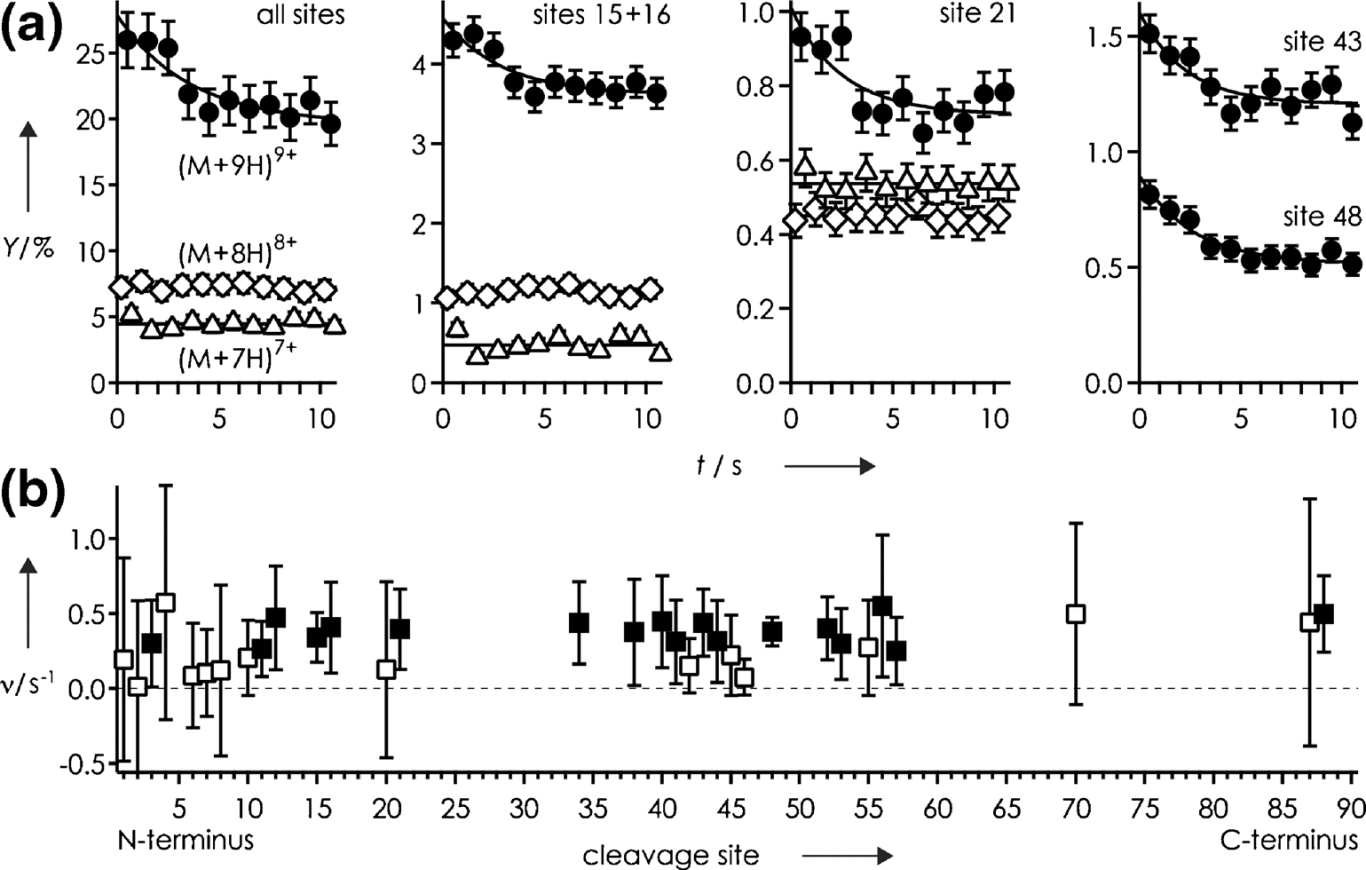
(**a**) Yield of separated ***c*** and ***z*** ^•^ fragments from ECD of (M + nH)^n+^ ions of KIX versus folding delay for n = 7 (triangles), n = 8 (diamonds), and n = 9 (circles) and sites as indicated; (**b**) exponential folding rates versus cleavage site, data points for which errors exceeded rates are highlighted as open symbols

The extent of unfolding after collisional activation is also reflected in the total yield of separated ***c*** and ***z***^•^ fragments, which increased with increasing (M + nH)^n+^ ion charge from ~5% for n = 7 to ~7% for n = 8 to ~26% for n = 9 (Figure 2a). Surprisingly, subsequent folding was observed only for the (M + 9H)^9+^ ions, for which the total yield of separated ***c*** and ***z***^•^ fragments decreased by ~6% to ~20% within 10 s, with an overall exponential folding rate of 0.282 ± 0.134 s^−1^ (Figure 2a, all sites). Within error limits (calculated as described in ref [34]), rates for individual sites were similar, but for some sites (e.g., 20, 42, 45, 46), errors exceeded rates (Figure 2b, open squares), indicating the possibility of no or far slower folding. Because these are scattered between sites indicating folding (Figure 2b, filled squares), it remains unclear if folding of the KIX (M + 9H)^9+^ ions is a global or a local process [34]. Even so, the overall folding rate of KIX (M + 9H)^9+^ ions is significantly higher than that of cytochrome *c* from tuna heart (0.067 ± 0.054 s^−1^), and comparable to the initial (up to ~4 s) folding rate of cytochrome *c* from horse heart [34]. These differences in folding rate roughly correlate with the proteins’ grand average of hydropathy (GRAVY) values [69], calculated by adding the hydropathy values of all amino acid residues and dividing by the number of residues in the sequence (see, for example, http://web.expasy.org/protparam/), that are similar for KIX (–0.901) and cytochrome *c* from horse heart (–0.902) but significantly smaller for cytochrome *c* from tuna heart (–0.727). This finding supports our previous hypotheses that the formation of electrostatic interactions such as salt bridges and hydrogen bonds is a major driving force for protein folding in the gas phase, and that more hydrophilic proteins generally fold faster [34]. Moreover, the similarity of site-specific rates (Figure 2b) suggests folding of the KIX (M + 9H)^9+^ ions into structures different from those without collisional activation (Figure 1e), as proposed previously [32].

**Figure 3:**
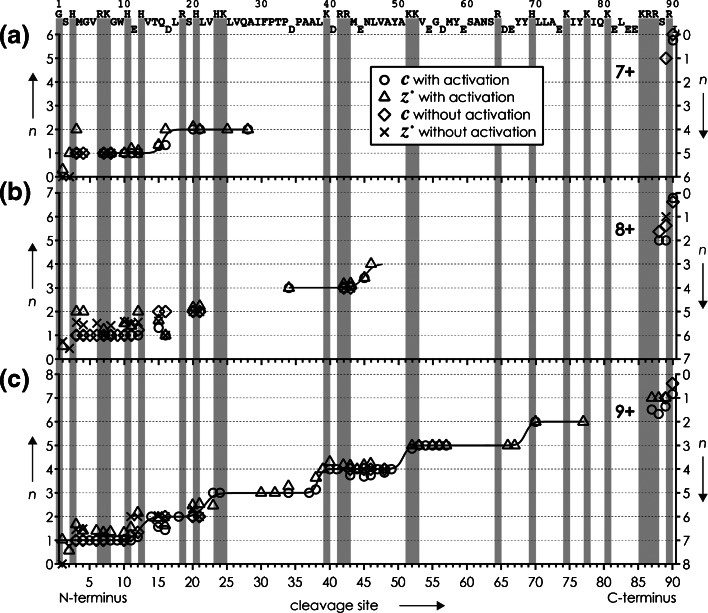
Average charge *n* of ***c*** (left axis) and complementary ***z*** ^•^ (right axis) fragments from ECD of (M + nH)^n+^ ions of KIX for (**a**) *n* = 7, (**b**) *n* = 8, and (**c**) *n* = 9, without and with 133, 128, and 90 eV laboratory frame energies for collisional activation prior to ECD, respectively, versus cleavage site. Gray bars indicate locations of basic residues, and solid lines are meant to guide the eye

Can the experimental observation that the KIX (M + nH)^n+^ ions with n = 7 and n = 8 did not fold on the timescale of the experiment, whereas folding was evident for those with n = 9, be attributed to Coulombic repulsion, despite the fact that increasing ion net charge should generally oppose protein folding? More precisely, could the net charge density and thus Coulombic repulsion in the unfolded regions of the (M + 7H)^7+^ and (M + 8H)^8+^ ions be higher than in the (M + 9H)^9+^ ions? The average charge of ***c*** and ***z***^•^ ions from ECD of (M + nH)^n+^ ions illustrated in Figure 3 immediately reveals that this is not the case, as the net charge of residues 1– 22 (cleavage site 22) is the same for (M + 7H)^7+^ and (M + 8H)^8+^ ions, and the net charge of residues 1–46 (cleavage site 46) is the same for (M + 8H)^8+^ and (M + 9H)^9+^ ions. Moreover, the average fragment charge values were similar with and without collisional activation prior to ECD, suggesting that unfolding of the KIX (M + nH)^n+^ ions by vibrational activation at the energies used here did not result in significant intramolecular proton mobilization [70].

However, the data in Figure 3 show that charge transitions of the (M + 9H)^9+^ ions were different from those of the (M + 7H)^7+^ and (M + 8H)^8+^ ions. For example, the transition from one to two charges for ***c*** ions (left axis, the corresponding transition for complementary ***z***^•^ ions is from four to five charges on the right axis) is around site 16 for (M + 7H)^7+^ ions but around site 13 for (M + 9H)^9+^ ions, and that from three to four charges is around site 45 for (M + 8H)^8+^ ions but around site 38 for (M + 9H)^9+^ ions. It is generally difficult to pinpoint the exact location of all charged sites in protein (M + nH)^n+^ ions from ECD data [30, 71] as capture of an electron neutralizes a positive charge, and because the presence of zwitterionic motifs that comprise both positively and negatively charged sites cannot be excluded. Moreover, even in unfolded structures, protons can be shared between adjacent residues in homodimeric (e.g., K⋅⋅⋅H^+^⋅⋅⋅K) or heterodimeric (e.g., K⋅⋅⋅H^+^⋅⋅⋅Q) ionic hydrogen bonds [51]. Nevertheless, the different charge transitions in Figure 3 clearly imply that charges are distributed differently in the 1–46 region for n = 7, 8 and n = 9. Because this is the only difference between the (M + 9H)^9+^ ions and the (M + 7H)^7+^ and (M + 8H)^8+^ ions in evidence, the propensity for folding must be related to the distribution of charges within the (M + nH)^n+^ ions.

**Figure 4:**
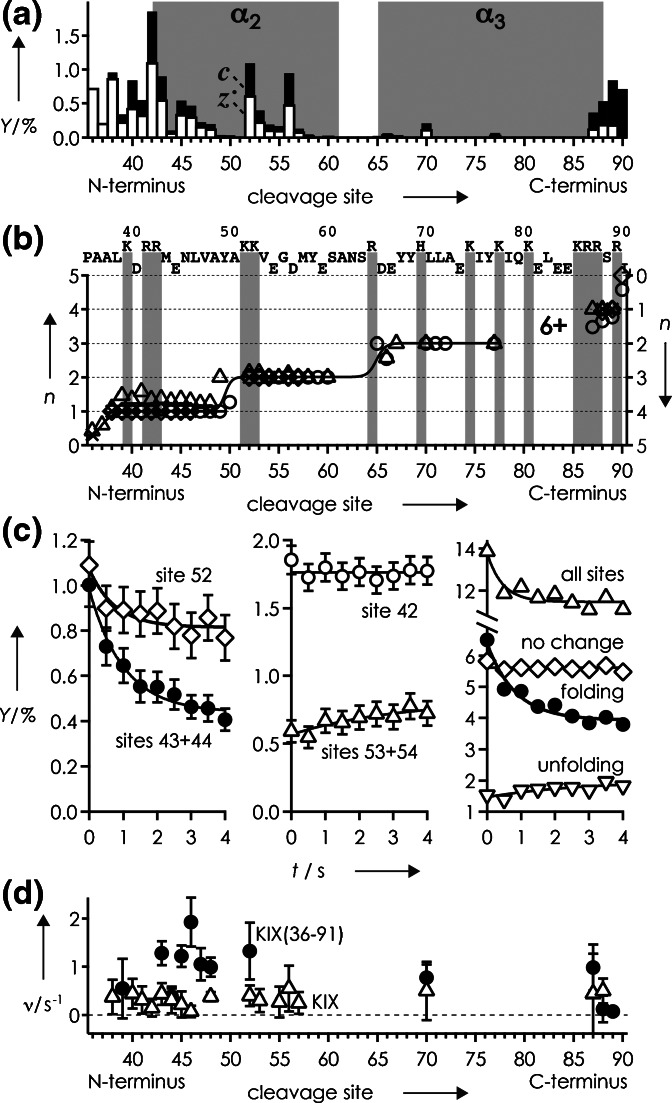
(**a**) yields of ***c*** (black bars) and ***z*** ^•^ (open bars) fragments from ECD of (M + 6H)^6+^ ions of KIX(36–91) after unfolding by collisional activation (90 eV), and (**b**) average charge *n* of ***c*** (left axis) and complementary ***z*** ^•^ (right axis) fragments, versus cleavage site with symbols as in Figure 3; (**c**) yield of ***c*** and ***z*** ^•^ fragments versus folding delay for sites as indicated (no change: sites 36, 37, 38, 40, 41, 42, 55, 57, 90; folding: sites 39, 43, 44, 45, 46, 47, 48, 52, 70, 87, 88, 89; unfolding: sites 53, 54, 56); (**d**) exponential folding rates versus cleavage site for KIX(36–91) (M + 6H)^6+^ ions (circles) and KIX (M + 9H)^9+^ ions (triangles)

To further test the hypothesis that the distribution of charges affects the propensity for folding, we have studied a peptide comprising residues 36–91 of KIX, termed KIX(36–91). ECD of collisionally activated (90 eV) KIX(36–91) (M + 6H)^6+^ ions gave a fragmentation pattern (Figure 4a) similar to that of KIX (M + 9H)^9+^ ions at 90 eV (Figure 1e), indicating that the secondary structure of helix α_3_ was largely retained in the peptide as well. Moreover, charge transitions occurred at nearly the same sites, around 38, 51, 68, and 90 for KIX, and 37, 50, 66, and 90 for KIX(36–91), as indicated by the average fragment charge values in Figure 4b. The overall folding rate of KIX(36–91) (M + 6H)^6+^ ions is 2.194 ± 0.930 s^−1^, and higher than that of KIX (M + 9H)^9+^ ions (0.282 ± 0.134 s^−1^) by a factor of ~8, which can be attributed to the more negative GRAVY value of KIX(36–91) of –1.118 and its shorter amino acid sequence that allows faster sampling of conformational space. Likewise, rates for individual sites are generally higher for KIX(36–91) (Figure 4d). Unlike for KIX, however, KIX(36–91) folding can unambiguously be classified as a local process driven by interactions between residues neighboring in sequence as sites indicating folding are separated by sites that show no change or even a slight increase in ECD fragment yield (Figure 4c), similar to the folding of cytochromes *c* [34]. Importantly, the fact that KIX(36–91) (M + 6H)^6+^ and KIX (M + 9H)^9+^ ions with similar charge distributions both show folding, whereas KIX (M + 7H)^7+^ and (M + 8H)^8+^ ions with different charge distributions do not, confirm our hypothesis that the distribution of charges within (M + nH)^n+^ ions is key to a gaseous ion's propensity for folding.

**Figure 5:**
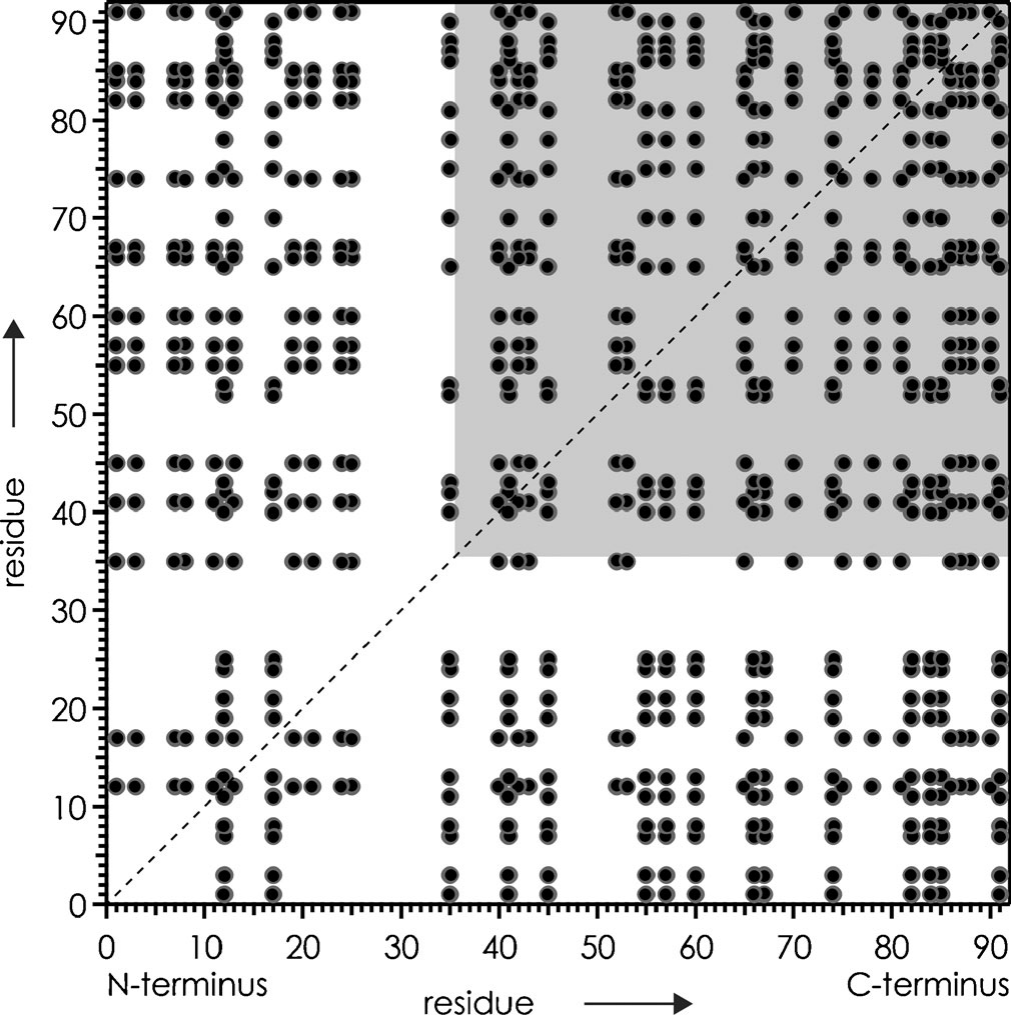
Potential salt bridges (not considering any structural restraints) between basic (H, K, R, N-terminus) and acidic (D, E, C-terminus) residues of KIX shown as cross peaks; gray square highlights region for KIX(36–91)

Not only do the KIX(36–91) (M + 6H)^6+^ and KIX (M + 9H)^9+^ ions fold at different rates, they also fold in different ways. Most strikingly, the region corresponding to cleavage sites 53–57 of KIX(36–91) shows no evidence for folding, whereas the same region of KIX does (Figure 4c, d). This difference in folding behavior can be attributed to the formation of electrostatic interactions, i.e., salt bridges and ionic or neutral hydrogen bonds [34] between residues in region 1–35 and 36–91 in the folding process of KIX that are not possible for KIX(36–91). For example, the formation of a salt bridge between D35 and R43 of KIX could explain the decrease in ECD fragment yield at sites 38, 40, 41, and 42 as an indicator for folding, and at the same time account for the lack of folding at these sites for KIX(36–91) that does not comprise D35. However, the number of salt bridges that could potentially form during folding of KIX, 360, and KIX(36–91), 180, is overall high (Figure 5), except in the regions corresponding to cleavage sites 1–10 and 17–33 (residues 1–11 and 17–34), for which KIX folding rates were generally exceeded by errors (Figure 2b) or could not be determined due to low ECD fragment ion abundances (Figure 1e). Because of the high number of potential interactions, it is difficult to rationalize the observed differences in folding behavior on the basis of specific interactions, such as salt bridges (Figure 5), between residues neighboring in sequence [34]. An additional complication in assigning potential interaction partners is the fact that neither KIX (M + 9H)^9+^ nor KIX(36–91) (M + 6H)^6+^ ions were fully unfolded after collisional activation (Figures 1e and 4a), which opens the possibility that interactions are formed between residues that are farther apart from each other in sequence, but close to each other in space.

Finally, we want to address the issue of disrupting and forming salt bridges in gaseous protein ions. Assuming, for example, that the native salt bridge between R19 and E55 is preserved in the (M + 7H)^7+^ ions of KIX, and broken by collisional activation, as indicated by the data in Figure 1c, does the latter involve charge separation? In other words, does breaking of the R19/E55 salt bridge produce protonated R19 and deprotonated E55 sidechains, or uncharged R19 and E55 sidechains? The proton affinity (PA) of guanidine as a sidechain model for arginine is 986 kJ/mol, and that of arginine is 1051 kJ/mol [72], with the 65 kJ/mol difference resulting from stabilization by intramolecular ionic hydrogen bonding in protonated arginine; the PA of propionate as a sidechain model for glutamate is 1454 kJ/mol [72]. PA values of pentane-1-amine and 4-methyl-1H-imidazole as sidechain models for lysine and histidine are somewhat lower than that of guanidine, 953 and 924 kJ/mol, respectively, and that of acetate as a sidechain model for aspartate is 1453 kJ/mol [72]. According to these PA values, proton transfer from a protonated basic sidechain to a deprotonated acidic sidechain is exothermic by 467–530 kJ/mol, which suggests that separation of residues in a salt bridge structure produces neutral sidechains unless the barrier between ionic (protonated basic sidechain and deprotonated acidic sidechain) and neutral forms (both sidechains uncharged) is sufficiently high to prevent proton transfer. Strittmatter and Williams have studied the energetics of heterodimers, AHB, consisting of trifluoroacetic acid, AH, and strong organic bases, B, and found by calculation that while the stability of the neutral (AH⋅B) and ion (A^−^⋅BH^+^) forms depends on the proton affinity of the base, barriers between the two forms were generally small [50]. Moreover, separation of the neutral pair AH⋅B into AH and B required only 82–99 kJ/mol, whereas separation of the ion pair A^−^⋅BH^+^ into A^−^ and BH^+^ required substantially more energy, 354–404 kJ/mol [50]. Activation barriers for interconversion of zwitterionic and non-zwitterionic structures of sodiated octaglycine, (GGGGGGGG + Na)^+^, were also small, between –0.25 and 1.25 kJ/mol [73]. It is thus reasonable to assume that by incrementally increasing an ion’s vibrational energy in low-energy collisional activation, a proton in a salt bridge structure will generally be transferred from the protonated basic sidechain to the deprotonated acidic sidechain before separation of the residues.

However, in compact protein ion structures like the KIX (M + nH)^n+^ ions investigated here, residues that form a salt bridge can at the same time be involved in additional electrostatic interactions with other residues that could substantially affect their proton affinity and the strength of a salt bridge. Previous studies have demonstrated the effect of inter- and intramolecular ionic and neutral hydrogen bonding on the proton affinity and related gas-phase basicity of neutral [74–77] and deprotonated [78–81] sites in amino acids and small peptides. In the native KIX structure, residue E55 not only forms a salt bridge with R19 but also an ionic hydrogen bond with Y68, and is in sufficiently close proximity to K52 to form yet another salt bridge (Figure 1b). If multiple electrostatic interactions can delocalize the negative charge of aspartate or glutamate residues, and thereby reduce their proton affinity to the extent that a positively charged, basic sidechain can be separated without causing proton transfer, remains an open question. Even so, the charge transition for the (M + 7H)^7+^ ions around site 16 (Figure 3a), near R19, is consistent with breaking of the R19/E55 salt bridge while retaining the positive and negative charge of R19 and E55, respectively; separation of protonated instead of uncharged R19 should also reduce the overall Coulombic repulsion in the partially unfolded ions. Regardless of whether or not proton transfer occurs upon breaking of salt bridges, the association of both neutral (e.g., R and E) and charged (e.g., protonated R and deprotonated E) pairs of basic and acidic sidechains can result in the formation of salt bridges as the barrier between zwitterionic (ion pair) and non-zwitterionic (neutral pair) structures is generally small [50, 73].

## Conclusion

We have studied the unfolding and folding of KIX (M + nH)^n+^ ions from native ESI by electron capture dissociation. Vibrational ion activation at energies that were sufficiently high to cause loss of small molecules such as NH_3_ and H_2_O by breaking of covalent bonds in about 5% of the KIX ions with n = 7–9 was insufficient for full unfolding, but high enough to break the native R19/E55 salt bridge in the (M + 7H)^7+^ ions. Apparently, the strength of a salt bridge between a protonated basic and a deprotonated acidic sidechain in a gaseous protein ion is similar to the strength of a covalent bond. Specifically, the native R19/E55 salt bridge provides strong stabilization of KIX’s tertiary structure after transfer into the gas phase by conjoining helices α_1_ and α_2_. In all KIX ions studied here, helix stability against vibrational activation increased from α_1_ to α_2_ to α_3_, which is the same order of stability as that in solution [62] and that found for nonactivated (M + nH)^n+^ ions with n = 7–16 [33]. This order of stability in the gas phase can be attributed to the number of stabilizing electrostatic interactions that increases from α_1_ to α_2_ to α_3_.

Folding of the KIX (M + nH)^n+^ ions on a 10 s timescale was observed only for n = 9, but not for n = 7 and n = 8, in contrast to what would be anticipated from Coulombic repulsion. Instead, the data for KIX and KIX(36–91) ions suggest that the propensity for protein folding in the gas phase is related to the distribution of charges within the (M + nH)^n+^ ions. Moreover, folding rates of different proteins showed a qualitative correlation with grand average of hydropathy (GRAVY) values, consistent with our previous hypothesis that the formation of electrostatic interactions, especially salt bridges, is a major driving force for protein folding in the gas phase [34]. Our finding that the propensity for folding is determined by the intramolecular distribution of charges instead of ion net charge challenges the widespread assumption that protein unfolding in the gas phase is generally caused by Coulombic repulsion, in agreement with a previous study of multiply protonated polypropylenamine dendrimers [82].
